# Education and stroke: evidence from epidemiology and Mendelian randomization study

**DOI:** 10.1038/s41598-020-78248-8

**Published:** 2020-12-03

**Authors:** Wen Xiuyun, Wu Qian, Xie Minjun, Li Weidong, Liao Lizhen

**Affiliations:** 1 Institute of Health, Guangdong Pharmaceutical University, Guangzhou, GuangDong People’s Republic of China; 2 Guangdong Engineering Research Center for Light and Health, Guangzhou Higher Education Mega Center, Guangdong Pharmaceutical University, Guangzhou, GuangDong People’s Republic of China; 3 Department of Psychology, Sun Yet-Sen University, Guangzhou, GuangDong People’s Republic of China; 4 The Second Clinical Medical College, Guangzhou University of Traditional Chinese Medicine, Guangzhou, GuangDong People’s Republic of China

**Keywords:** Genetics, Neurology

## Abstract

We aim to characterize the association between education and incident stroke (including total stroke, ischemic stroke, and hemorrhagic stroke) and assess whether there is a causal relationship between them. The final sample size was 11,509 in this study from the Atherosclerosis Risk in Communities (ARIC) study. Cox hazard regression models were used to explore the association between education level and incident stroke. Two-sample Mendelian randomization (MR) was used to estimate the causality. During a median follow-up of 25.3 years, 915 cases (8.0%) of stroke occurred. Participants with advanced education level were associated with 25% (HR 0.75; 95% CI 0.62, 0.91) decreased the rate of incident total stroke. Hazard ratio of intermediate and advanced education level for ischemic stroke were 0.82 (0.69, 0.98) and 0.73 (0.60, 0.90) separately. In the MR analysis, we observed evidence that education was likely a negetive causal risk factor for ischemic stroke (OR 0.764, 95% CI 0.585–0.998, *P* = 0.048). Higher education level was associated with a decreased rate of total stroke and ischemic stroke incident, but not hemorrhagic stroke incident. There might be a protective causal association between education and ischemic stroke (but not total stroke nor hemorrhagic stroke).

## Introduction

Stroke is the second leading cause of death and disability globally^1,2^. In Europe every year, more than 1.5 million people suffer a stroke^3^. As stroke is related to substantially increased morbidity, mortality, and economic costs, identifying unknown, potentially modifiable risk factors is essential, which is likely to improve the outcome of stroke risk patients. Growing evidence supported that low education was associated with increased stroke risk^4,5^, independent of many traditional risk factors. However, such associations could be affected by confounding from unmeasured or unknown factors and were therefore not reliable for inferring causality^6^. On the other hand, it was not possible to conduct randomized controlled trials to study the effects of education on stroke^7^. Mendelian randomization (MR) was an epidemiological study design. It used genetic variants associated with the modifiable risk factor as proxy indicators to establish causal effect on outcomes^8^. It could reduce confounding by environmental factors because alleles were randomly allocated at conception. Moreover, it also avoided reverse causation bias because disease could not affect genotype^9^. Recently, a two-sample MR analysis reported that education protected against ischaemic stroke risk independently of cognition^10^, indicating there was a causal role of genetically determined short education on the increased risk of ischaemic stroke. Yet the nature and magnitude of the prospective association between education and incident stroke in a population-based cohort with a long follow-up period remained to be unclear. Whatsmore, we still did not know if there was a causal relationship between education and risk of stroke, especially the hemorrhagic type.

At this moment, we aim to characterize the nature and magnitude of the prospective association between education and incident stroke in a population-based cohort-the Atherosclerosis Risk in Communities (ARIC) Study reliably. Also, we use the MR method to assess whether there is a causal relationship between education and risk of different types of strokes, including total stroke, ischemic stroke, and hemorrhagic stroke.

## Results

### Baseline characteristics

Baseline characteristics were shown in Table 1. Of 11,509 participants, 2,475 individuals were with basic education level, 4785 with intermediate level, and 4249 with an advanced level. Participants with low education levels were more likely to be black, smoker, higher body mass index (BMI), higher systolic blood pressure (SBP), higher diastolic blood pressure (DBP), and so on.

**Table 1:** Baseline characteristic of study populations by education levels.

Characteristic	Total (n = 11,509)	Basic (n = 2,475)	Intermediate (n = 4,785)	Advanced (n = 4,249)	P value
Age, years	54.0 ± 5.7	55.9 ± 5.6	53.8 ± 5.6	53.2 ± 5.7	< 0.001
Male	5182 (45.0%)	1126 (45.5%)	1901 (39.7%)	2155 (50.7%)	< 0.001
Black	2634 (22.9%)	1077 (43.5%)	740 (15.5%)	817 (19.2%)	< 0.001
Smoking					< 0.001
Never	4846 (42.1%)	908 (36.7%)	2024 (42.3%)	1914 (45.0%)	
Former	3676 (31.9%)	732 (29.6%)	1456 (30.4%)	1488 (35.0%)	
Current	2987 (26.0%)	835 (33.7%)	1305 (27.3%)	847 (19.9%)	
Drinking					< 0.001
Never	2738 (23.8%)	824 (33.3%)	1147 (24.0%)	767 (18.1%)	
Former	2038 (17.7%)	722 (29.2%)	793 (16.6%)	523 (12.3%)	
Current	6733 (58.5%)	929 (37.5%)	2845 (59.5%)	2959 (69.6%)	
BMI, kg/m^2^	27.4 ± 5.2	28.5 ± 5.9	27.3 ± 5.2	26.9 ± 4.7	< 0.001
SBP, mmHg	120.6 ± 18.5	125.9 ± 20.1	120.2 ± 18.3	118.2 ± 17.1	< 0.001
DBP, mmHg	73.4 ± 11.1	75.0 ± 12.1	72.9 ± 10.8	72.9 ± 10.7	< 0.001
Sport	2.46 ± 0.8	2.22 ± 0.7	2.43 ± 0.8	2.63 ± 0.8	< 0.001
Creatinine, mg/dl	1.1 ± 0.4	1.1 ± 0.5	1.1 ± 0.4	1.1 ± 0.3	< 0.001
HDL-c, mmol/L	1.4 ± 0.4	1.3 ± 0.4	1.3 ± 0.4	1.4 ± 0.4	0.031
LDL-c, mmol/L	3.5 ± 1.0	3.6 ± 1.1	3.6 ± 1.0	3.5 ± 1.0	< 0.001
TC, mmol/L	5.5 ± 1.1	5.6 ± 1.1	5.6 ± 1.1	5.4 ± 1.0	< 0.001
TG, mmol/L	1.4 ± 0.7	1.4 ± 0.7	1.4 ± 0.7	1.3 ± 0.7	< 0.001
Glucose, mmol/L	5.9 ± 2.0	6.3 ± 2.7	5.9 ± 2.0	5.7 ± 1.5	< 0.001
PLT, 10^3^/mm^3^	257.9 ± 64.7	254.9 ± 67.5	260.0 ± 64.8	257.4 ± 63.0	0.006
Diabetes	932 (8.1%)	347 (14.0%)	344 (7.2%)	241 (5.7%)	< 0.001
Stain	51 (0.4%)	9 (0.4%)	24 (0.5%)	18 (0.4%)	0.683
Aspirin	5262 (45.7%)	1014 (41.0%)	2300 (48.1%)	1948 (45.8%)	< 0.001
Anticoagulants	32 (0.3%)	16 (0.6%)	8 (0.2%)	8 (0.2%)	< 0.001

BMI = body mass index; SBP = systolic blood pressure; DBP = diastolic blood pressure; HDL-c = high-density lipoprotein cholesterol; LDL-c = low-density lipoprotein cholesterol; TC = total cholesterol; TG = triglyceride; PLT = Platelet count.

Education was categorized as basic education (less than high school completion), intermediate education (high school degree or vocational school) and advanced education (attending or completed college or professional school).

### Education and incident stroke

**Figure 1:**
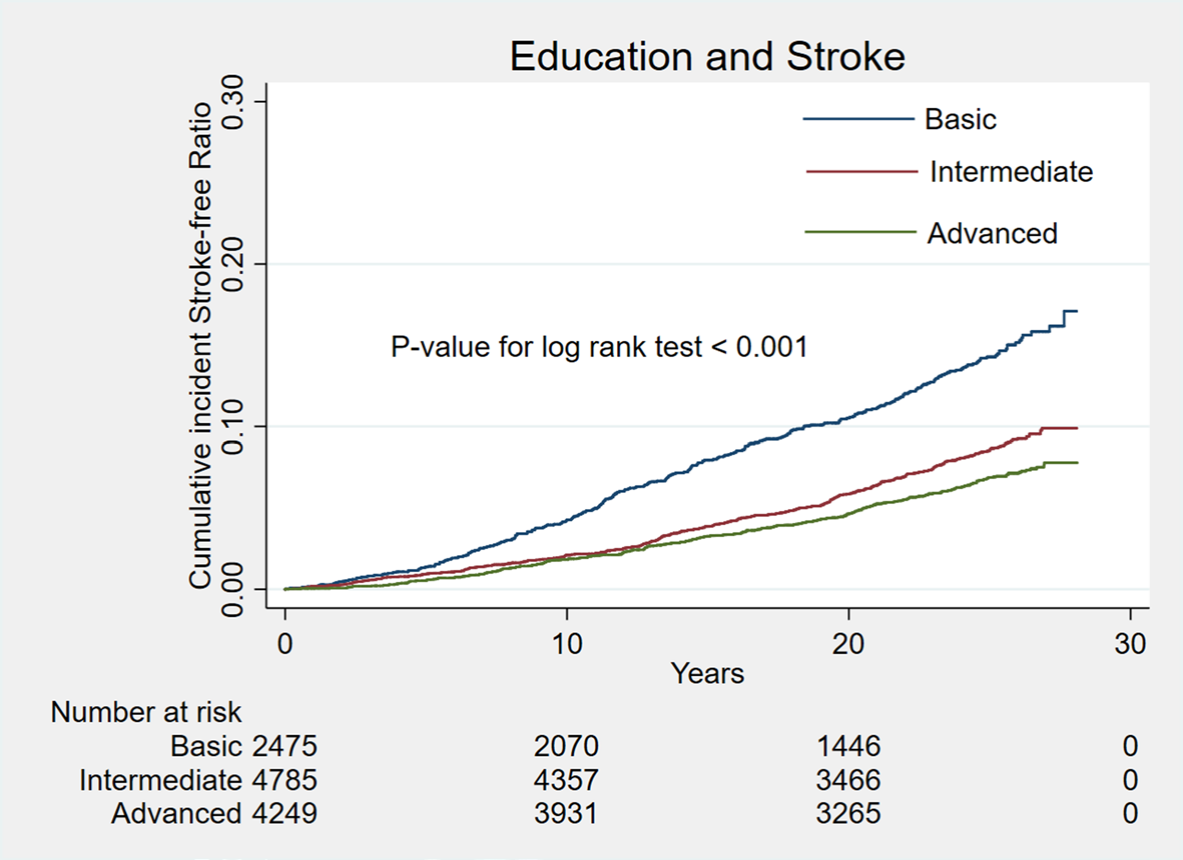
Unadjusted cumulative curves for incident stroke. During a median follow-up of 25.3 years, 915 cases (8.0%) of stroke occurred. Unadjusted cumulative curves for incident total stroke were shown. Education was categorized as basic education (less than high school completion), intermediate education (high school degree or vocational school) and advanced education (attending or completed college or professional school).

**Figure 2:**
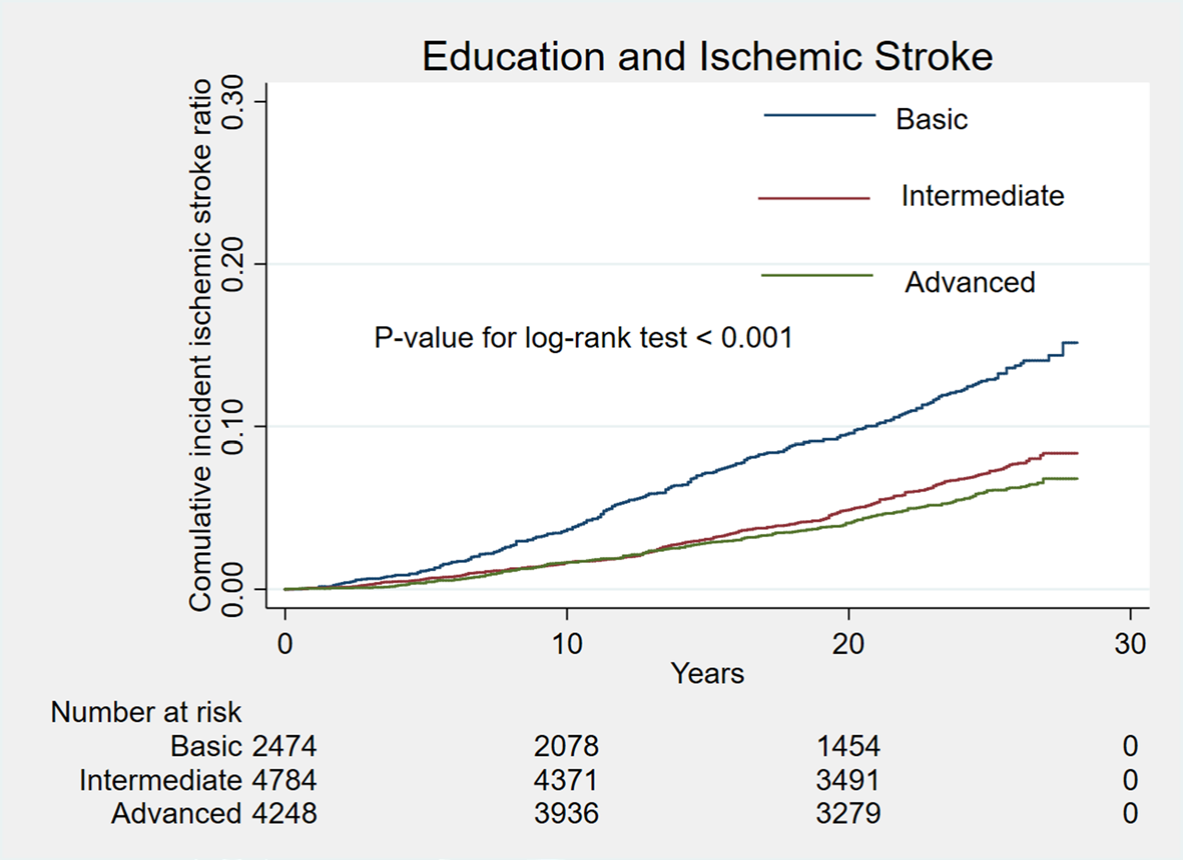
Unadjusted cumulative curves for incident ischemic stroke. Unadjusted cumulative curves for incident ischemic stroke were shown. Education was categorized as basic education (less than high school completion), intermediate education (high school degree or vocational school) and advanced education (attending or completed college or professional school).

**Figure 3:**
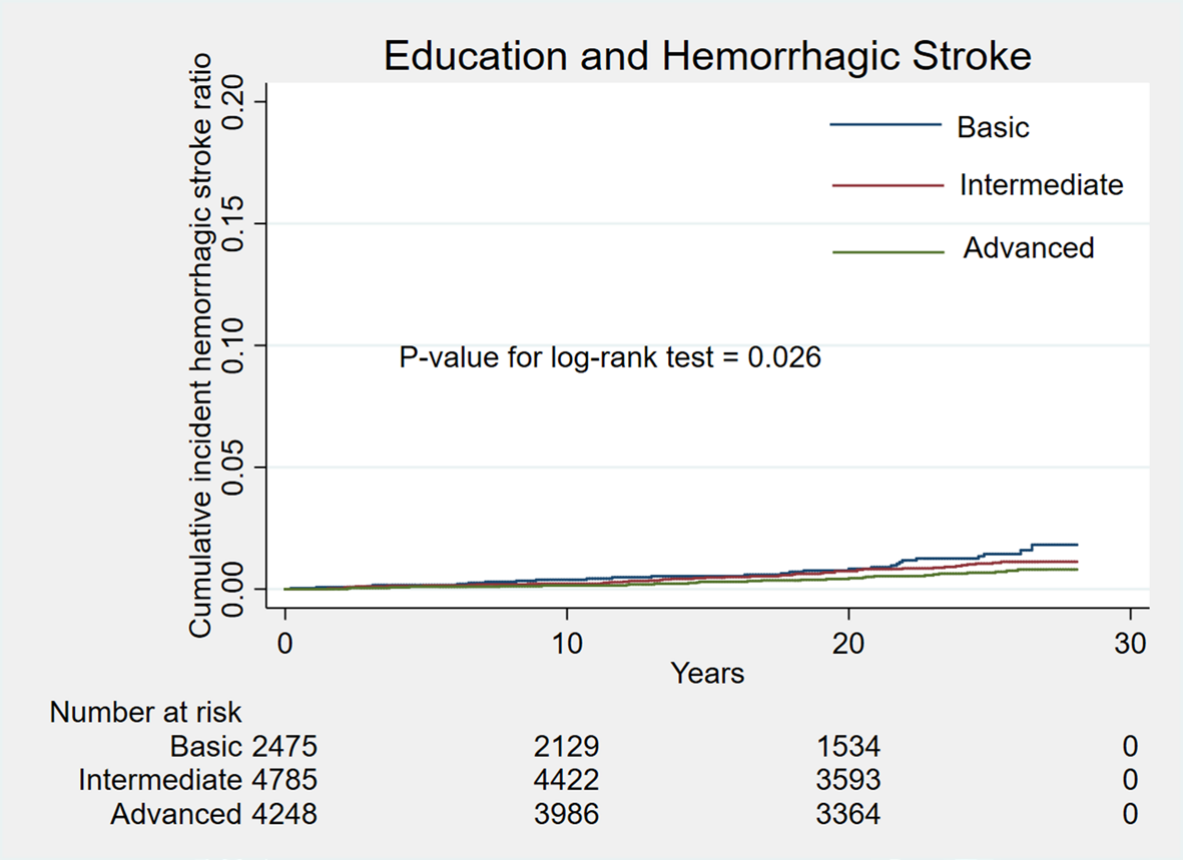
Unadjusted cumulative curves for incident hemorrhagic stroke. Unadjusted cumulative curves for incident hemorrhagic stroke were shown. Education was categorized as basic education (less than high school completion), intermediate education (high school degree or vocational school) and advanced education (attending or completed college or professional school).

During a median follow-up of 25.3 years, 915 cases (8.0%) of stroke occurred. Unadjusted cumulative curves for incident total stroke were shown in Fig. 1. Participants with basic education had the highest risk of incident stroke (P for log-rank test < 0.001). In the second model, after adjusting for a series of traditional cardiovascular risk factors, participants with intermediate and advanced education level were associated with 17% (HR 0.83; 95% CI 0.71, 0.98) decreased and 33% (HR 0.67; 95% CI 0.56, 0.80) decreased rate of incident stroke separately (Table 2). After further adjusting for family income in the final model, participants with advanced education levels were still associated with 25% (HR 0.75; 95% CI 0.62, 0.91) decreased rate of incident stroke (Table 2). Unadjusted cumulative curves for incident ischemic stroke and hemorrhagic stroke were shown in Figs. 2 and 3. Participants with basic education had the highest risk of incident ischemic stroke (P for log-rank test < 0.001) and hemorrhage stroke (P for log-rank test = 0.026). The hazard ratio of intermediate and advanced education level for ischemic stroke in the final model were 0.82 (0.69, 0.98) and 0.73 (0.60, 0.90) separately. For hemorrhagic stroke, hazard ratios of intermediate and advanced education level were 1.23 (0.73, 2.06) and 0.98 (0.54, 1.80) separately (Table 2).

**Table 2:** Hazard ratio for education in different types of strokes.

Type	Events rate	Model 1		Model 2		Model 3	
		HR (95% CI)	P value	HR (95% CI)	P value	HR (95% CI)	P value
**Stroke**							
Basic	287/2,475	Reference	–	Reference	–	Reference	–
Intermediate	364/4,785	0.80 (0.68, 0.94)	0.006	0.83 (0.71, 0.98)	0.031	0.88 (0.74, 1.04)	0.135
Advanced	264/4,249	0.61 (0.51, 0.72)	< 0.001	0.67 (0.56, 0.80)	< 0.001	0.75 (0.62, 0.91)	0.004
**Ischemic stroke**							
Basic	258/2,475	Reference	–	Reference	–	Reference	–
Intermediate	306/4,785	0.74 (0.62, 0.88)	0.001	0.79 (0.66, 0.94)	0.007	0.82 (0.69, 0.98)	0.032
Advanced	231/4,249	0.59 (0.49, 0.71)	< 0.001	0.66 (0.55, 0.80)	< 0.001	0.73 (0.60, 0.90)	0.003
**Hemorrhagic stroke**							
Basic	27/2,475	Reference	–	Reference	–	Reference	–
Intermediate	43/4,785	1.11 (0.67, 1.85)	0.691	1.11 (0.67, 1.85)	0.691	1.23 (0.73, 2.06)	0.439
Advanced	27/4,249	0.73 (0.42, 1.26)	0.256	0.73 (0.42, 1.28)	0.272	0.98 (0.54, 1.80)	0.955

Model 1: Adjusted for age, race, gender; Model 2: Further adjusted for creatine, HDL-c, LDL-c, TG, glucose, PLT, diabetes, Stain, Aspirin, Anticoagulant drug; Model 3: Further adjusted for income. Education was categorized as basic education (less than high school completion), intermediate education (high school degree or vocational school) and advanced education (attending or completed college or professional school).

HDL-c = high-density lipoprotein cholesterol; LDL-c = low-density lipoprotein cholesterol; TG = triglyceride; PLT = Platelet count.

**Figure 4:**
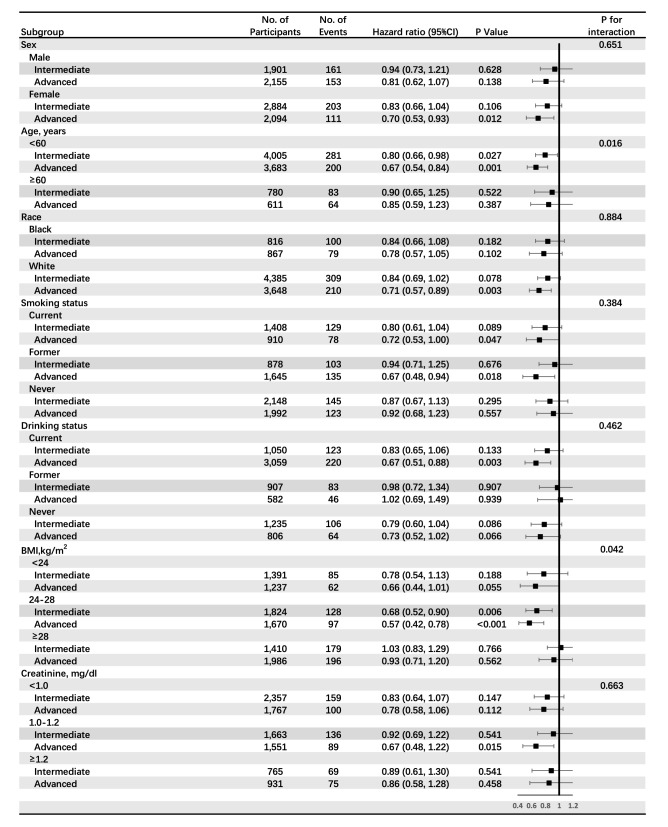
Subgroups analyses of the association between education and incident stroke. Pre-specified subgroups by sex, age, race, smoking, drinking, BMI and creatine were analyzed. Education was categorized as basic education (less than high school completion), intermediate education (high school degree or vocational school) and advanced education (attending or completed college or professional school). BMI, Body mass index.

In subgroup analyses, results were similar when stratified by sex, race, smoking, drinking, BMI, and creatine (all P-interactions > 0.1, Fig. 4). However, hazard ratios of education level were significant for participants with moderate BMI category 0.68 (0.52, 0.90) for intermediate level and 0.57 (0.42, 0.78) for advanced level, and P-value for interaction was 0.042. Besides, hazard ratios of education level were 0.80 (0.66, 0.98) and 0.67 (0.54, 0.84) for participants with education level of intermediate and advanced separately in the subgroup of age < 60 years old and 0.90 (0.65, 1.25) and 0.85 (0.59, 1.23) in subgroup of age ≥ 60 years old. The *P* value for interaction between subgroups of age and education level was 0.016 (Fig. 4). We speculated that both BMI and age affected the causality between education and stroke. The protective effect of education on stroke existed in the BMI (range 24–28) category and age < 60 years old group.

To sum up, our study supported that participants with higher education levels were associated with a decreased rate of total stroke and ischemic stroke incident, but not hemorrhagic stroke incident.

### Causal associations between genetically determined education and different types of stroke

**Figure 5:**
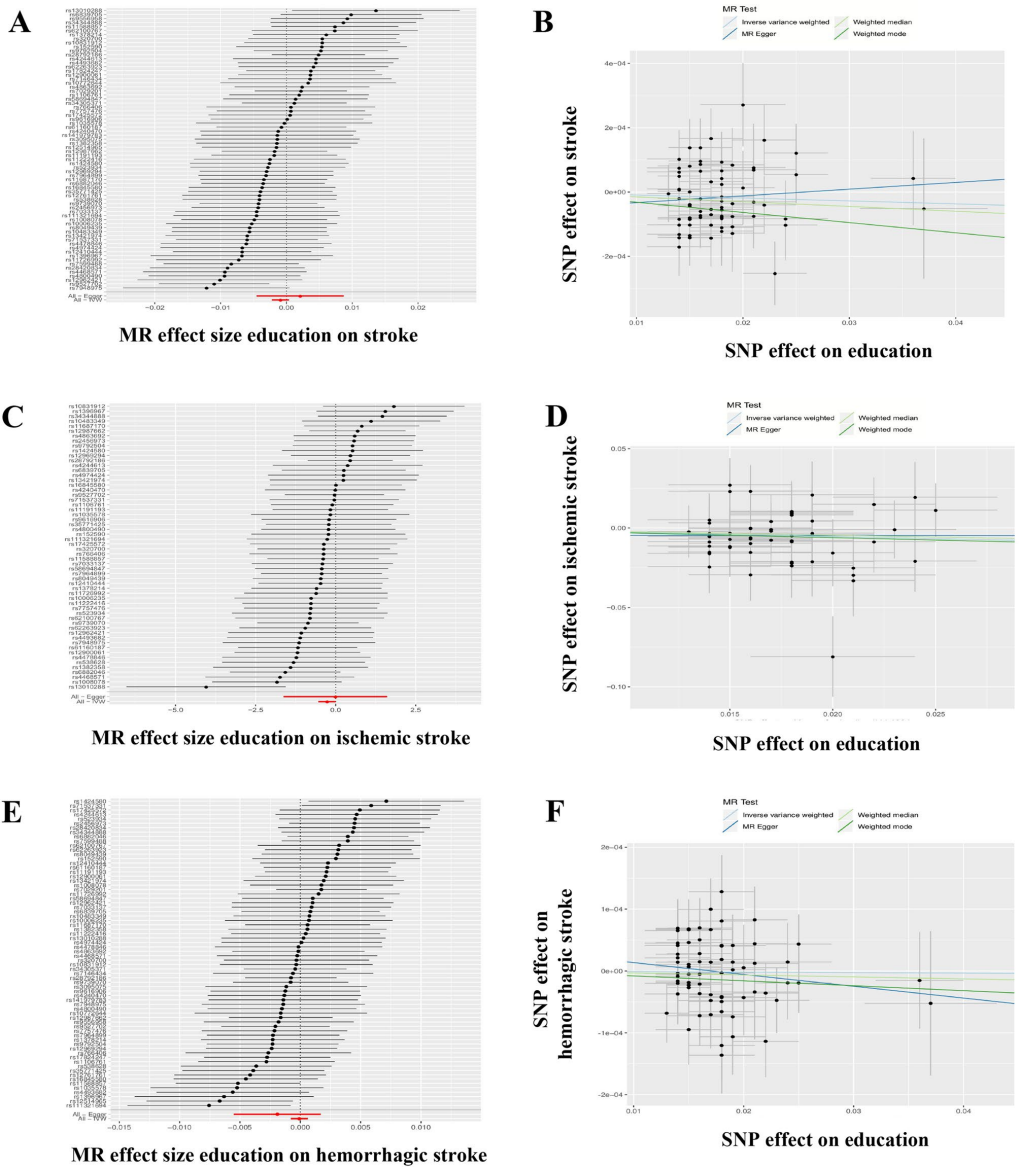
Forest plots (**A**, **C**, **E**) and scatter plots (**B**, **D**, **F**) of causal effects between education‐associated SNPs and risk of stroke/ischemic stroke/hemorrhagic stroke. The slopes of each line in the scatter plot represent the causal association for each method. SNP, single-nucleotide polymorphism; MR, Mendelian randomization; IVW, inverse‐variance weighted.

The 70 education-associated SNPs explained 1.04% of the variance in education level, and the mean F-statistic of the instruments was 68, suggesting adequate strength. In the Education-Stroke MR analysis by using the conventional method (IVW), we did not detect a causal relation between them (OR 0.999, 95% CI 0.998–1.000, P = 0.166). We detected no heterogeneity between estimates from individual SNPs (P_heterogeneity_ = 0.616 [MR-Egger] and P_heterogeneity_ = 0.621 [IVW]) nor pleiotropy effect (P_pleiotropy_ = 0.368) (Table 3, Fig. 5B). We found no single instrument was strongly driving the overall effect of Education-Stroke in the leave-one-out analysis (Figure Supplement 1A). Besides, there was no funnel plot asymmetry (Figure Supplement 1B). So the leave-one-out analysis and funnel plot further both suggested that no SNPs exhibited horizontal pleiotropy. Moreover, results from the weighted median, Weighted mode, and MR Egger method were consistent (all P > 0.05) (Table 3, Fig. 5A).

**Table 3:** Causal associations between genetically determined education and different types of strokes.

Exposure-outcome	Method	Causal estimate				
		SNP	OR	95% CI		P value
Education-Stroke	MR Egger	70	1.002	0.995	1.009	0.539
	Weighted median	70	0.999	0.997	1.001	0.144
	IVW	70	0.999	0.998	1.000	0.166
	Weighted mode	70	0.997	0.992	1.002	0.198
Test for Heterogeneity: P = 0.616 (MR-Egger) and P = 0.621 (IVW)						
Test for Horizontal pleiotropy: MR-Egger intercept = -0.000054, se = 0.000059, P = 0.368						
Education-Ischemic stroke	MR Egger	58	0.990	0.196	4.992	0.991
	Weighted median	58	0.796	0.547	1.157	0.233
	IVW	58	0.764	0.585	0.998	0.048
	Weighted mode	58	0.738	0.320	1.705	0.480
Test for Heterogeneity: P = 0.917 (MR-Egger) and P = 0.923 (IVW)						
Test for horizontal pleiotropy: MR-Egger intercept = -0.0045, se = 0.014, P = 0.751						
Education-Hemorrhagic stroke	MR Egger	70	0.998	0.998	0.995	0.302
	Weighted median	70	1.000	1.000	0.999	0.539
	IVW	70	1.000	1.000	0.999	0.811
	Weighted mode	70	0.999	0.999	0.997	0.508
Test for heterogeneity: P = 0.411 (MR-Egger) and P = 0.410 (IVW)						
Test for horizontal pleiotropy: MR-Egger intercept = 0.000033, se = 0.000032, P = 0.315						

SNP, single-nucleotide polymorphism; OR, odds ratio; CI, confidence interval; IVW, Inverse variance weighted.

When Education-Hemorrhagic stroke was analyzed, the genetically determined education was also not causally associated with hemorrhagic stroke (all P > 0.05) (Table 3, Fig. 5E,F) either. The leave-one-out analysis and funnel plot further also suggested that no SNPs exhibited horizontal pleiotropy (Figure Supplement 1E,F). To sum up, our MR study indicated no causal associations between genetically determined education and total stroke nor hemorrhagic stroke.

However, when Education-Ischemic stroke was analyzed, as shown in Table 3, in conventional MR analysis by inverse-variance weighted (IVW) method, we observed evidence that education was likely a negetive causal risk factor for ischemic stroke (odds ratio [OR] 0.764, 95% CI 0.585–0.998, P = 0.048) with no evidence of heterogeneity between estimates from individual SNPs (P_heterogeneity_ = 0.917 [MR-Egger] and P_heterogeneity_ = 0.923 [IVW]) nor pleiotropy effect (P_pleiotropy_ = 0.751) (Table 3; Fig. 5C,D), though the weighted median, weighted mode, and MR Egger method were not detected significantly differently. Since the leave-one-out analysis and funnel plot further suggested that no SNPs exhibited horizontal pleiotropy (Figure Supplement 1C,D), our MR study supported that there might be a protective causal association between education and ischemic stroke.

## Discussion

In this large-scale, population-based prospective cohort without pre-existing stroke, we showed that participants with higher education were associated with a decreased rate of total stroke and ischemic stroke incident, but not hemorrhagic stroke incident. Furthermore, utilizing large-scale summary genome-wide association studies (GWAS) data, our MR study supported that there might be a protective causal association between education and ischemic stroke (but not total stroke nor hemorrhagic stroke).

Stroke had emerged as a significant public health priority in the whole world in recent decades. It was the second most common cause of death and a common cause of dependency and dementia. Nevertheless, stroke was preventable, for those with modifiable risk factors can be targeted for primary prevention of stroke. The 10 top modifiable risk factors for stroke were hypertension, lack of physical activity, abnormal lipids, unhealthy diet, abdominal obesity, psychological factors, current smoking, cardiac causes, alcohol consumption, and diabetes^11^. Together, these 10 risk factors accounted for close to 90% risk of stroke^11^. Moreover, much epidemiological evidence showed that low education levels were linked to the stroke incident^12–14^. A recent prospective cohort study with a mean follow-up time of 4.7 years in 253 657 participants also revealed that low education was associated with increased stroke risk in both sexes^15^. Similarly, a Meta-analysis reported that having < 11 versus ≥ 11 years of education was associated with an increase in relative risk of stroke of about a third^16^. In our study, with a median follow-up of 25.3 years in 11,509 participants, advanced education level participants were associated with 25% (HR 0.75; 95% CI 0.62, 0.91) decreased the rate of incident stroke, which was in accordance with the previous observational association researches. We considered that with > 20 years of follow-up and more than 10,000 participants, the negative association between education and stroke which was detected in the well-designed study-the ARIC study, might represent the real situation. We had added more convincing evidence supporting the protective role of education on stroke.

Total stroke was subtyped into two main kinds-the ischemic and hemorrhagic stroke. Since their mechanisms were quite different, we expected that education might have different effects on these two types of strokes. Meanwhile, we were curious about whether there was a causal relationship between education and risk of total stroke and its subtypes. The protective role of educational attainment on cardiovascular disease (CVD), including stroke, had been described in previous observational work^17,18^, as well as MR studies^10,19^. A recent MR study revealed that BMI, systolic blood pressure, and smoking behavior mediated a substantial proportion of the protective effect of education on the risk of cardiovascular (coronary heart disease, stroke, myocardial infarction, and cardiovascular disease) outcomes^20^. The explanation for why educational attainment protected against stroke might relate to the broad benefits of education. For example, higher educational attainment was associated with a healthier lifestyle, an occupation with safer working conditions, and better access to health care^21,22^. Yet our MR study indicated no causal associations between genetically determined education and total stroke nor hemorrhagic stroke. At the same time, education was likely a negetive causal risk factor for ischemic stroke. We analyzed that it might be explained that stroke was a heterogeneous collection of clinically related but distinct disorders, meaning that different subtypes had distinct underlying pathologies^23,24^. Take the serum lipids, for example, contrasting relationships with serum lipids were found in ischemic and hemorrhagic stroke^25,26^. High levels of total cholesterol (TC) and LDL (low-density lipoprotein) cholesterol (LDL-C) were both associated with an increased risk of ischemic stroke, whereas low levels were associated with an increased risk of hemorrhagic stroke^27^. Also, a meta-analysis of 185 genome-wide association studies showed that 1 SD of genetically elevated LDL-C was associated with an increased risk of ischemic stroke (OR 1.12; 95% CI 1.04–1.20), but not hemorrhagic stroke^28^. LDL-C reduction agents were reported reducing incident ischemic stroke, suggesting a causal relationship between LDL-C reduction and ischemic stroke prevention^29^. Yet a signal of increased risk for hemorrhagic stroke in the SPARCL trial and nonsignificant increases in risk in IMPROVE-IT, FOURIER, and the Cholesterol Treatment Trialists meta-analysis suggested that marked lowering of LDL-C could cause hemorrhagic strokes^29^. We considered that it might due to that cholesterol had long been recognized as a risk factor for atherosclerosis in the pathogen of ischemic stroke but not hemorrhagic stroke. Since ischemic stroke representing 70–90% of all strokes^23,24^, and a higher level of education, bringing a lot of other benefits besides ischemic stroke. The critical importance of education should be reflected in public health and educational policy and governmental decision-making for many disease prevention.

There are several limitations of this study. First, there are many other confounders that we should but can not adjust since they are not measured in the ARIC study at visit 1, such as many inflammatory markers (e.g. BNP, hsCRP) which will affect the atherosclerosis progress and ends up in impacting stroke. Second, in the ARIC cohort, the ascertainment of stroke needs to be improved. Incident stroke is identified from annual telephone interviews, visits, or surveillance of community hospitals. As a result, the ascertainment of stroke in ARIC may miss patients who suffer the transient ischaemic attack, leading to a lower stroke incidence. Third, we performed our MR study on the MR-Base platform. SSGAC consortium for education and MRC-IEU consortium for stroke were the data that we could get on the platform. Larger GWAS data should be used to confirm the causal association between education and stroke. Last but not least, both SSGAC consortium and MRC-IEU consortium contain only European populations. Hence, our findings in a European people cannot readily be generalized to the population with different ethnic and racial backgrounds.

To sum up, a higher education level was associated with a decreased rate of total stroke and ischemic stroke incident, but not hemorrhagic stroke incident in the ARIC study. There was likely a protective causal association between education and ischemic stroke (but not total stroke nor hemorrhagic stroke).

## Methods

This study was approved by the ethics committee of Guangdong Pharmaceutical University. All methods were performed in accordance with the relevant guidelines and regulations of Declaration of Helsinki.

### Study population

The ARIC Study is a population-based, prospective cohort study of cardiovascular risk factors in four US communities. The cohort included 15,792 participants aged 45–64 years between 1987 and 1989 (visit 1), and four subsequent study visits were conducted in 1990–1992 (visit 2), 1993–1995 (visit 3), 1996–1998 (visit 4), and 2011–2013 (visit 5). Participants are also follow-up by annual or semiannual telephone interviews and active surveillance of community hospitals. The ARIC Study has been approved by institutional review boards at all institutions, and all participants provided written informed consent. The detailed study design has been described previously^30^. In the present study, we excluded participants with missing data of follow-up for stroke (n = 279), participants with previous stroke (n = 266), participants with previous CHD or HF (n = 1062), participants with missing data of education (n = 15), and participants with missing other covariates (n = 2661). The final sample size was 11,509.

### Education assessment

Education was self-reported at visit 1 as the highest grade completed in school and was categorized as basic education (less than high school completion), intermediate education (high school degree or vocational school) and advanced education (attending or completed college or professional school).

### Stroke ascertainment

Incident stroke was ascertained through 2014 from visit 1. Detail ascertainment of incident stroke has been described previously^31,32^. Stroke was defined as rapid onset of a focal neurological deficit lasting > 24 h or until death in the absence of a non-stroke cause, including ischemic and hemorrhagic stroke. A stroke event was identified from annual telephone interviews, visits or surveillance of community hospitals. A stroke event was considered in hospitalization if the discharge diagnosis contained an International Classification of Diseases-9th Revision (ICD-9) code of 430 to 438 or discharged summary included a keyword related to cerebrovascular procedure or disease, or if there was imaging evidence of cerebrovascular disease.

### Measurement of other covariates

Covariates were assessed at visit 1. Race, gender, age, smoking, drinking and family income were self-reported. Height and weight were measured with the participants wearing light clothes, and body mass index was calculated as weight (in kilograms) divided by squared height (in meters). Blood pressure was obtained in seated participants by certified technicians using a random-zero sphygmomanometer. The sport was accessed using the validated Baecke questionnaire. Diabetes was defined as fasting blood glucose ≥ 126 mg/dl, non-fasting blood glucose ≥ 200 mg/dl, use of antidiabetic medicine, or self-reported physician diagnosis of diabetes. Stroke, coronary heart disease (CHD), and heart failure (HF) at baseline were defined as previously^33–35^. Stroke was defined by self-reported. CHD was defined as physician-diagnosed CHD or presence of a previous myocardial infraction by ECG. HF was defined as the reported use of medicines to treat heart failure in the previous 2 weeks or the presence of heart failure according to Gothenburg criteria^33–35^. Total cholesterol, high-density lipoprotein cholesterol (HDL-c), and triglycerides were measured using standardized enzymatic assays and low-density lipoprotein (LDL-c) was calculated based on the Friedewald formula. Creatine was measured using a modified kinetic Jaffe method^30^.

### Genetic association analysis by MR

We performed MR in a strategy known as two-sample MR based on publicly available summary-level data from GWAS to estimate the causal associations between genetically determined education and different types of strokes, including total stroke, ischemic stroke, and hemorrhagic stroke^36^. We conducted our MR study on the MR-Base platform online (http://www.mrbase.org)^37^. As instrumental variables for the MR analyses, we selected all SNPs associated with education at genome-wide significance (P < 5 × 10^–8^) in the available GWAS (Table Supplement 1). We extracted the following data for each genetic instrument for traits from GWAS of the following outcomes: the effect allele (EA), effect allele frequency (EAF), Beta value, standard error (SE), SNP, and P-value. We also requested the following metrics of SNP genotype quality from disease and risk factor studies: strong evidence of between-study heterogeneity in the SNP-trait association (P ≤ 0.001), Hardy–Weinberg disequilibrium (P ≤ 0.001) or imputation quality metric (info or r^2^) ≤ 0.90. We harmonized the summary data for diseases and risk factors so that the effect allele reflected the allele associated with exposure. When SNPs were palindromic, i.e., A/T or G/C, we used the information on allele frequency to resolve strand ambiguity. We excluded SNP-trait associations from the GWAS catalog if they were missing a P-value, Beta, or a SE for the Beta. Proxy SNPs were not included in analyses. Summary statistics from the SSGAC consortium (Pubmed ID 27225129, European, males and females, sample size 293,723) were used for education, MRC-IEU consortium data was used for stroke (ID UKB-b:6813, European, males and females, sample size 463,010), ISGC consortium data was used for ischemic stroke (Pubmed ID 26935894, mixed, males and females, sample size 29,633), MRC-IEU consortium data was used for hemorrhagic stroke (ID UKB-b:4538, mixed, males and females, sample size 463,010). Details of studies and datasets used for analyses were presented in Table Supplement 2.

### Statistical analysis

Baseline characteristics of participants between education levels were compared using 1-way ANOVA test, the χ^2^ test, and the Kruskal–Wallis test, as appropriate. Kaplan–Meier estimate was used to compute cumulative incidents of incident stroke by education levels and the difference in assessment was compared using the log-rank test. We used Cox hazard regression models to explore the association between education level and incident stroke. Time of follow-up was defined as the time from visit 1 to outcomes, loss to follow-up, death, or 31 December 2014, whichever occurred first. The initial model adjusted for age, race and gender. The second model further adjusted for diabetes, creatine, HDL-c, LDL-c, triglyceride, glucose, blood platelet, use of statin, aspirin, and anticoagulant drug. The final model further adjusted for income. In order to explore the potential heterogeneity of the association between education and stroke, pre-specified subgroup analyses were performed by gender, age, race, smoking status, drinking status, BMI, and creatine. We investigated the interaction by fitting the variables*education term to the final model and P for interaction was examined by the likelihood ratio test. We would accept a P-value of > 0.1 to determine results that are similar between subgroups. Cox hazard regression models were also used to assess the association between education level and ischemic stroke and hemorrhagic stroke separately.

For MR analysis, the association of each SNP with education was weighted by its association with total stroke, ischemic stroke, and hemorrhagic stroke, and estimates were combined using an IVW method^38^. Several sensitivity analyses were carried out, including (1) the leave-one-out analysis, in which one SNP, in turn, was removed to evaluate the impact of outlying SNPs; (2) funnel plots, in which the estimate for a particular SNP was plotted against its precision, and asymmetry in the funnel plot indicated violations of the assumption through horizontal pleiotropy; (3) the weighted median method, which gives accurate estimates if at least 50% of the instrumental variables are valid; (4) the weighted mode method, which clusters the SNPs into groups based on the similarity of causal effects, and returns the causal effect estimate based on the cluster that has the largest number of SNPs^39^, and (5) MR-Egger regression, which can detect and adjust for pleiotropy^40^. The strength of the instrumental variables was assessed using the F-statistic. All reported odds ratios (ORs) with their 95% confidence intervals (CIs) are scaled to 1 SD (3.6 years) increase in education. All statistical tests were 2-sided. The threshold of statistical significance for the analyses of 58 or 70 SNPs by Bonferroni correction was P < 0.0009 or 0.0007 ([P < 0.05]/58 SNPs, [P < 0.05]/70 SNPs,); statistical test for the MR analyses of three types of strokes were considered statistically significant at P < 0.05.

## Supplementary information

Supplementary Figure 1.

Supplementary Information 2.

Supplementary Table 1.

Supplementary Table 2.
