# Supplementary figures and images for: Education and stroke: evidence from epidemiology and Mendelian randomization study
Source: Sci Rep. 2020 Dec 3;10:21208. doi: 10.1038/s41598-020-78248-8 (PMC7713498; doi:10.1038/s41598-020-78248-8)

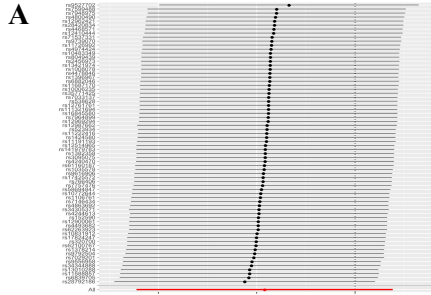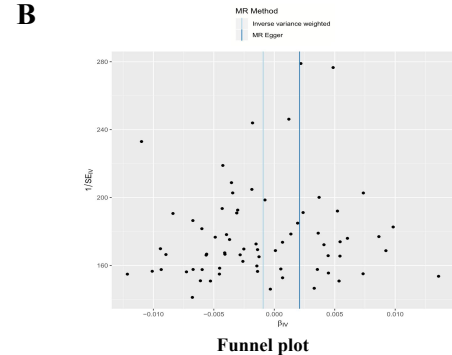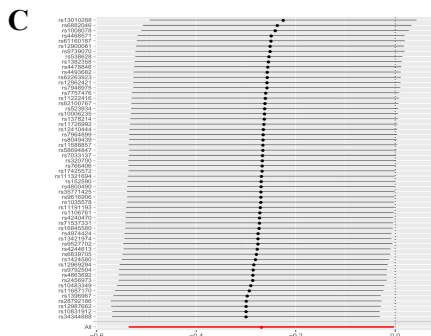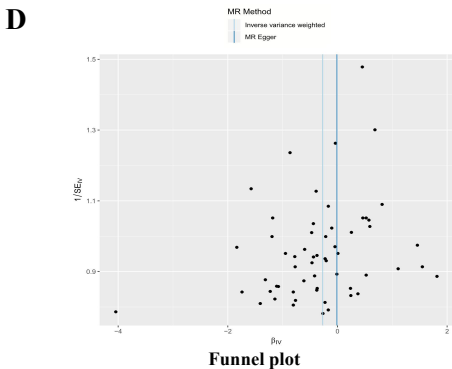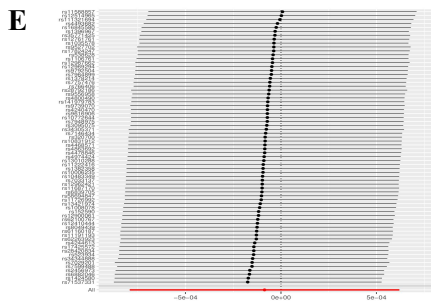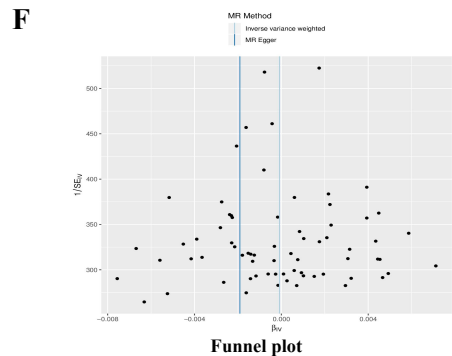

Supplement: Supplementary file 1 — Supplementary Figure 1. [file 41598_2020_78248_MOESM1_ESM.pdf]
