# Supplementary material for: Education and stroke: evidence from epidemiology and Mendelian randomization study
Source: Sci Rep. 2020 Dec 3;10:21208. doi: 10.1038/s41598-020-78248-8 (PMC7713498; doi:10.1038/s41598-020-78248-8)
Supplement: Supplementary file 2 — Supplementary Information 2. [file 41598_2020_78248_MOESM2_ESM.doc]

supplementary information

**Education and stroke: evidence from epidemiology and Mendelian randomization study**

Wen Xiuyun1,2; Wu Qian3,4; Xie Minjun4; Li Weidong1,2; Liao Lizhen1,2

1 Institute of Health, Guangdong Pharmaceutical University, Guangzhou GuangDong, PR China

2 Guangdong Engineering Research Center for Light and Health, Guangzhou Higher Education Mega Center, Guangzhou, GuangDong, P.R. China

3 Department of Psychology, Sun Yet-sen University; Guangzhou GuangDong, PR China; The Second Clinical Medical College，Guangzhou University of Traditional Chinese Medicine, Guangzhou GuangDong, PR China

4 The Second Clinical Medical College, Guangzhou University of Traditional Chinese Medicine, Guangzhou GuangDong, PR China

**Running title:** Education and stroke

Authors contributed equally to the study: Wen Xiuyun and Wu Qian

Corresponding to: Li Weidong (gylwd26@163.com) and Liao Lizhen (liaolizhen2013gy@163.com), Guangdong Pharmaceutical University, Guangzhou Higher Education Mega Center, Guangzhou, GuangDong, P.R. China.

**Figure Supplement 1** Leave-one-out sensitivity analysis and funnel plot. (A, C, E) Leave-one-out sensitivity analysis. Each black point represents the IVW MR method applied to estimate the causal effect of education on stroke/ischemic stroke/hemorrhagic stroke, excluding that particular variant from the analysis. The red point depicts the IVW estimate using all SNPs. There are no instances where the exclusion of one particular SNP leads to dramatic changes in the overall result. (B, D, F) Funnel plot of the relationship between the causal effect of education on stroke/ischemic stroke/hemorrhagic stroke. Funnel plot showing the relationship between the causal effect of education on stroke/ischemic stroke/hemorrhagic stroke estimated using each SNP as a separate instrument against the inverse of the standard error of the causal estimate. Vertical lines show the causal estimates using all SNPs combined into a single instrument for the two different methods. Asymmetry in the funnel plot may be indicative of violations of the assumption through horizontal pleiotropy.
