# Supplementary material for: Education and stroke: evidence from epidemiology and Mendelian randomization study
Source: Sci Rep. 2020 Dec 3;10:21208. doi: 10.1038/s41598-020-78248-8 (PMC7713498; doi:10.1038/s41598-020-78248-8)
Supplement: Supplementary file 3 — Supplementary Table 1. [file 41598_2020_78248_MOESM3_ESM.docx]

**Table Supplement 1 Characteristics of the SNPs associated with education and different types of strokes**

| SNP | EA | Other | EAF | Associations with Education | | |  | Associations with Stroke | | |
| --- | --- | --- | --- | --- | --- | --- | --- | --- | --- | --- |
|  |  | allele |  | Beta | se | P value |  | Beta | se | P value |
| rs10006235 | T | C | 0.287 | -0.015 | 0.003 | 0.000 |  | 0.000 | 0.000 | 0.610 |
| rs1008078 | T | C | 0.373 | -0.016 | 0.003 | 0.000 |  | 0.000 | 0.000 | 0.710 |
| rs1035578 | A | G | 0.569 | -0.013 | 0.002 | 0.000 |  | 0.000 | 0.000 | 0.008 |
| rs10483349 | A | G | 0.830 | -0.019 | 0.003 | 0.000 |  | 0.000 | 0.000 | 0.400 |
| rs10772644 | C | G | 0.871 | 0.021 | 0.004 | 0.000 |  | 0.000 | 0.000 | 0.710 |
| rs10831912 | T | C | 0.403 | -0.015 | 0.003 | 0.000 |  | 0.000 | 0.000 | 0.750 |
| rs1106761 | A | G | 0.360 | -0.017 | 0.003 | 0.000 |  | 0.000 | 0.000 | 0.830 |
| rs11130222 | A | T | 0.577 | 0.026 | 0.003 | 0.000 |  | 0.000 | 0.000 | 0.089 |
| rs111321694 | T | C | 0.183 | -0.018 | 0.003 | 0.000 |  | 0.000 | 0.000 | 0.220 |
| rs11191193 | A | G | 0.651 | 0.019 | 0.003 | 0.000 |  | 0.000 | 0.000 | 0.660 |
| rs11222416 | T | C | 0.416 | -0.015 | 0.003 | 0.000 |  | 0.000 | 0.000 | 0.023 |
| rs11588857 | A | G | 0.209 | 0.022 | 0.003 | 0.000 |  | 0.000 | 0.000 | 0.810 |
| rs11687170 | T | C | 0.828 | 0.024 | 0.004 | 0.000 |  | 0.000 | 0.000 | 0.560 |
| rs11726992 | T | C | 0.646 | 0.014 | 0.003 | 0.000 |  | 0.000 | 0.000 | 0.790 |
| rs12410444 | A | G | 0.718 | -0.018 | 0.003 | 0.000 |  | 0.000 | 0.000 | 0.090 |
| rs12514965 | T | C | 0.739 | 0.018 | 0.003 | 0.000 |  | 0.000 | 0.000 | 0.120 |
| rs12534506 | A | T | 0.453 | -0.015 | 0.003 | 0.000 |  | 0.000 | 0.000 | 0.700 |
| rs12761761 | T | C | 0.207 | 0.017 | 0.003 | 0.000 |  | 0.000 | 0.000 | 0.190 |
| rs12900061 | A | G | 0.162 | 0.021 | 0.003 | 0.000 |  | 0.000 | 0.000 | 0.890 |
| rs12962421 | A | G | 0.537 | -0.014 | 0.002 | 0.000 |  | 0.000 | 0.000 | 0.760 |
| rs12969294 | A | G | 0.379 | -0.018 | 0.003 | 0.000 |  | 0.000 | 0.000 | 0.610 |
| rs12987662 | A | C | 0.379 | 0.022 | 0.003 | 0.000 |  | 0.000 | 0.000 | 0.520 |
| rs13010288 | T | G | 0.112 | 0.020 | 0.004 | 0.000 |  | 0.000 | 0.000 | 0.004 |
| rs13421974 | T | C | 0.522 | 0.014 | 0.002 | 0.000 |  | 0.000 | 0.000 | 0.620 |
| rs1378214 | T | C | 0.383 | -0.016 | 0.003 | 0.000 |  | 0.000 | 0.000 | 0.350 |
| rs1382358 | T | C | 0.907 | 0.021 | 0.004 | 0.000 |  | 0.000 | 0.000 | 0.520 |
| rs1396967 | T | C | 0.606 | -0.015 | 0.003 | 0.000 |  | 0.000 | 0.000 | 0.570 |
| rs141979783 | T | C | 0.052 | 0.037 | 0.006 | 0.000 |  | 0.000 | 0.000 | 0.710 |
| rs1424580 | T | C | 0.800 | 0.018 | 0.003 | 0.000 |  | 0.000 | 0.000 | 0.630 |
| rs152590 | C | G | 0.343 | 0.014 | 0.003 | 0.000 |  | 0.000 | 0.000 | 0.740 |
| rs16845580 | T | C | 0.631 | 0.016 | 0.003 | 0.000 |  | 0.000 | 0.000 | 0.700 |
| rs17425572 | A | G | 0.440 | 0.014 | 0.002 | 0.000 |  | 0.000 | 0.000 | 0.340 |
| rs17824247 | T | C | 0.580 | -0.018 | 0.003 | 0.000 |  | 0.000 | 0.000 | 0.920 |
| rs2456973 | A | C | 0.679 | -0.018 | 0.003 | 0.000 |  | 0.000 | 0.000 | 0.260 |
| rs28420834 | A | G | 0.429 | -0.015 | 0.003 | 0.000 |  | 0.000 | 0.000 | 0.860 |
| rs28792186 | T | C | 0.605 | -0.025 | 0.003 | 0.000 |  | 0.000 | 0.000 | 0.370 |
| rs3095075 | A | G | 0.550 | -0.014 | 0.002 | 0.000 |  | 0.000 | 0.000 | 0.900 |
| rs320700 | A | G | 0.634 | 0.016 | 0.003 | 0.000 |  | 0.000 | 0.000 | 0.300 |
| rs34305371 | A | G | 0.088 | 0.036 | 0.004 | 0.000 |  | 0.000 | 0.000 | 0.340 |
| rs34344888 | A | G | 0.397 | -0.016 | 0.003 | 0.000 |  | 0.000 | 0.000 | 0.490 |
| rs35771425 | T | C | 0.795 | 0.019 | 0.003 | 0.000 |  | 0.000 | 0.000 | 0.560 |
| rs4240470 | C | G | 0.707 | 0.016 | 0.003 | 0.000 |  | 0.000 | 0.000 | 0.082 |
| rs4244613 | A | G | 0.416 | -0.014 | 0.003 | 0.000 |  | 0.000 | 0.000 | 0.650 |
| rs4468571 | A | G | 0.577 | -0.014 | 0.003 | 0.000 |  | 0.000 | 0.000 | 0.420 |
| rs4478846 | T | C | 0.853 | 0.018 | 0.003 | 0.000 |  | 0.000 | 0.000 | 0.840 |
| rs4493682 | C | G | 0.203 | 0.019 | 0.003 | 0.000 |  | 0.000 | 0.000 | 0.240 |
| rs4800490 | A | C | 0.543 | -0.015 | 0.002 | 0.000 |  | 0.000 | 0.000 | 0.044 |
| rs4863692 | T | G | 0.334 | 0.018 | 0.003 | 0.000 |  | 0.000 | 0.000 | 0.440 |
| rs4974424 | A | G | 0.827 | -0.019 | 0.003 | 0.000 |  | 0.000 | 0.000 | 0.310 |
| rs523934 | A | G | 0.416 | 0.015 | 0.003 | 0.000 |  | 0.000 | 0.000 | 0.580 |
| rs538628 | C | G | 0.244 | -0.018 | 0.003 | 0.000 |  | 0.000 | 0.000 | 0.100 |
| rs58694847 | C | G | 0.310 | -0.018 | 0.003 | 0.000 |  | 0.000 | 0.000 | 0.660 |
| rs61160187 | A | G | 0.619 | -0.018 | 0.003 | 0.000 |  | 0.000 | 0.000 | 0.210 |
| rs62100767 | A | G | 0.601 | 0.014 | 0.003 | 0.000 |  | 0.000 | 0.000 | 0.047 |
| rs62263923 | A | G | 0.644 | -0.016 | 0.003 | 0.000 |  | 0.000 | 0.000 | 0.490 |
| rs6839705 | A | C | 0.360 | 0.017 | 0.003 | 0.000 |  | 0.000 | 0.000 | 0.300 |
| rs6882046 | A | G | 0.687 | -0.021 | 0.003 | 0.000 |  | 0.000 | 0.000 | 0.360 |
| rs7029201 | A | G | 0.424 | 0.025 | 0.003 | 0.000 |  | 0.000 | 0.000 | 0.480 |
| rs7033137 | C | G | 0.774 | 0.016 | 0.003 | 0.000 |  | 0.000 | 0.000 | 0.340 |
| rs7146434 | A | G | 0.565 | -0.014 | 0.002 | 0.000 |  | 0.000 | 0.000 | 0.056 |
| rs71537331 | T | C | 0.371 | -0.017 | 0.003 | 0.000 |  | 0.000 | 0.000 | 0.230 |
| rs7599488 | T | C | 0.425 | -0.017 | 0.002 | 0.000 |  | 0.000 | 0.000 | 0.680 |
| rs766406 | T | G | 0.619 | 0.014 | 0.003 | 0.000 |  | 0.000 | 0.000 | 0.600 |
| rs7757476 | A | G | 0.231 | 0.020 | 0.003 | 0.000 |  | 0.000 | 0.000 | 0.830 |
| rs7948975 | T | C | 0.659 | 0.014 | 0.003 | 0.000 |  | 0.000 | 0.000 | 0.420 |
| rs7964899 | A | G | 0.457 | 0.017 | 0.002 | 0.000 |  | 0.000 | 0.000 | 0.094 |
| rs8049439 | T | C | 0.655 | 0.015 | 0.003 | 0.000 |  | 0.000 | 0.000 | 0.250 |
| rs9527702 | A | G | 0.763 | 0.023 | 0.003 | 0.000 |  | 0.000 | 0.000 | 0.140 |
| rs9556958 | T | C | 0.502 | -0.015 | 0.002 | 0.000 |  | 0.000 | 0.000 | 0.390 |
| rs9616906 | A | G | 0.452 | 0.015 | 0.003 | 0.000 |  | 0.000 | 0.000 | 0.130 |
| rs9739070 | A | G | 0.230 | 0.024 | 0.003 | 0.000 |  | 0.000 | 0.000 | 0.390 |
| rs9792504 | A | G | 0.694 | -0.018 | 0.003 | 0.000 |  | 0.000 | 0.000 | 0.340 |
| SNP | EA | Other | EAF | Associations with Education | | |  | Associations with Ischemic stroke | | |
|  |  | allele |  | Beta | se | P value |  | Beta | se | P value |
| rs10006235 | T | C | 0.287 | -0.015 | 0.003 | 0.000 |  | 0.012 | 0.018 | 0.528 |
| rs1008078 | T | C | 0.373 | -0.016 | 0.003 | 0.000 |  | 0.029 | 0.017 | 0.075 |
| rs1035578 | A | G | 0.569 | -0.013 | 0.002 | 0.000 |  | 0.002 | 0.016 | 0.894 |
| rs10483349 | A | G | 0.830 | -0.019 | 0.003 | 0.000 |  | -0.021 | 0.021 | 0.316 |
| rs10831912 | T | C | 0.403 | -0.015 | 0.003 | 0.000 |  | -0.027 | 0.017 | 0.107 |
| rs1106761 | A | G | 0.360 | -0.017 | 0.003 | 0.000 |  | 0.002 | 0.017 | 0.914 |
| rs11130222 | A | T | 0.577 | 0.026 | 0.003 | 0.000 |  | -0.011 | 0.017 | 0.494 |
| rs111321694 | T | C | 0.183 | -0.018 | 0.003 | 0.000 |  | 0.005 | 0.023 | 0.835 |
| rs11191193 | A | G | 0.651 | 0.019 | 0.003 | 0.000 |  | -0.003 | 0.018 | 0.854 |
| rs11222416 | T | C | 0.416 | -0.015 | 0.003 | 0.000 |  | 0.012 | 0.016 | 0.480 |
| rs11588857 | A | G | 0.209 | 0.022 | 0.003 | 0.000 |  | -0.009 | 0.020 | 0.660 |
| rs11687170 | T | C | 0.828 | 0.024 | 0.004 | 0.000 |  | 0.020 | 0.022 | 0.377 |
| rs11726992 | T | C | 0.646 | 0.014 | 0.003 | 0.000 |  | -0.009 | 0.016 | 0.592 |
| rs12410444 | A | G | 0.718 | -0.018 | 0.003 | 0.000 |  | 0.009 | 0.018 | 0.632 |
| rs12900061 | A | G | 0.162 | 0.021 | 0.003 | 0.000 |  | -0.025 | 0.021 | 0.232 |
| rs12962421 | A | G | 0.537 | -0.014 | 0.002 | 0.000 |  | 0.015 | 0.016 | 0.352 |
| rs12969294 | A | G | 0.379 | -0.018 | 0.003 | 0.000 |  | -0.008 | 0.017 | 0.627 |
| rs12987662 | A | C | 0.379 | 0.022 | 0.003 | 0.000 |  | 0.015 | 0.017 | 0.373 |
| rs13010288 | T | G | 0.112 | 0.020 | 0.004 | 0.000 |  | -0.081 | 0.025 | 0.001 |
| rs13421974 | T | C | 0.522 | 0.014 | 0.002 | 0.000 |  | 0.003 | 0.016 | 0.841 |
| rs1378214 | T | C | 0.383 | -0.016 | 0.003 | 0.000 |  | 0.010 | 0.017 | 0.563 |
| rs1382358 | T | C | 0.907 | 0.021 | 0.004 | 0.000 |  | -0.030 | 0.026 | 0.253 |
| rs1396967 | T | C | 0.606 | -0.015 | 0.003 | 0.000 |  | -0.023 | 0.016 | 0.158 |
| rs1424580 | T | C | 0.800 | 0.018 | 0.003 | 0.000 |  | 0.009 | 0.020 | 0.643 |
| rs152590 | C | G | 0.343 | 0.014 | 0.003 | 0.000 |  | -0.003 | 0.017 | 0.853 |
| rs16845580 | T | C | 0.631 | 0.016 | 0.003 | 0.000 |  | 0.000 | 0.017 | 0.997 |
| rs17425572 | A | G | 0.440 | 0.014 | 0.002 | 0.000 |  | -0.005 | 0.016 | 0.752 |
| rs2456973 | A | C | 0.679 | -0.018 | 0.003 | 0.000 |  | -0.010 | 0.017 | 0.548 |
| rs28792186 | T | C | 0.605 | -0.025 | 0.003 | 0.000 |  | -0.011 | 0.017 | 0.505 |
| rs320700 | A | G | 0.634 | 0.016 | 0.003 | 0.000 |  | -0.006 | 0.017 | 0.722 |
| rs34344888 | A | G | 0.397 | -0.016 | 0.003 | 0.000 |  | -0.023 | 0.016 | 0.157 |
| rs35771425 | T | C | 0.795 | 0.019 | 0.003 | 0.000 |  | -0.004 | 0.019 | 0.830 |
| rs4240470 | C | G | 0.707 | 0.016 | 0.003 | 0.000 |  | 0.000 | 0.018 | 0.989 |
| rs4244613 | A | G | 0.416 | -0.014 | 0.003 | 0.000 |  | -0.005 | 0.017 | 0.757 |
| rs4468571 | A | G | 0.577 | -0.014 | 0.003 | 0.000 |  | 0.024 | 0.017 | 0.141 |
| rs4478846 | T | C | 0.853 | 0.018 | 0.003 | 0.000 |  | -0.022 | 0.021 | 0.299 |
| rs4493682 | C | G | 0.203 | 0.019 | 0.003 | 0.000 |  | -0.021 | 0.022 | 0.341 |
| rs4800490 | A | C | 0.543 | -0.015 | 0.002 | 0.000 |  | 0.003 | 0.016 | 0.833 |
| rs4863692 | T | G | 0.334 | 0.018 | 0.003 | 0.000 |  | 0.011 | 0.018 | 0.546 |
| rs4974424 | A | G | 0.827 | -0.019 | 0.003 | 0.000 |  | -0.005 | 0.023 | 0.841 |
| rs523934 | A | G | 0.416 | 0.015 | 0.003 | 0.000 |  | -0.012 | 0.019 | 0.515 |
| rs538628 | C | G | 0.244 | -0.018 | 0.003 | 0.000 |  | 0.024 | 0.021 | 0.248 |
| rs58694847 | C | G | 0.310 | -0.018 | 0.003 | 0.000 |  | 0.008 | 0.019 | 0.678 |
| rs61160187 | A | G | 0.619 | -0.018 | 0.003 | 0.000 |  | 0.021 | 0.017 | 0.213 |
| rs62100767 | A | G | 0.601 | 0.014 | 0.003 | 0.000 |  | -0.011 | 0.017 | 0.496 |
| rs62263923 | A | G | 0.644 | -0.016 | 0.003 | 0.000 |  | 0.015 | 0.017 | 0.366 |
| rs6839705 | A | C | 0.360 | 0.017 | 0.003 | 0.000 |  | 0.004 | 0.017 | 0.797 |
| rs6882046 | A | G | 0.687 | -0.021 | 0.003 | 0.000 |  | 0.033 | 0.019 | 0.073 |
| rs7033137 | C | G | 0.774 | 0.016 | 0.003 | 0.000 |  | -0.007 | 0.018 | 0.711 |
| rs71537331 | T | C | 0.371 | -0.017 | 0.003 | 0.000 |  | 0.001 | 0.018 | 0.964 |
| rs766406 | T | G | 0.619 | 0.014 | 0.003 | 0.000 |  | -0.005 | 0.017 | 0.749 |
| rs7757476 | A | G | 0.231 | 0.020 | 0.003 | 0.000 |  | -0.016 | 0.021 | 0.461 |
| rs7948975 | T | C | 0.659 | 0.014 | 0.003 | 0.000 |  | -0.016 | 0.017 | 0.346 |
| rs7964899 | A | G | 0.457 | 0.017 | 0.002 | 0.000 |  | -0.008 | 0.016 | 0.646 |
| rs8049439 | T | C | 0.655 | 0.015 | 0.003 | 0.000 |  | -0.007 | 0.016 | 0.666 |
| rs9527702 | A | G | 0.763 | 0.023 | 0.003 | 0.000 |  | -0.001 | 0.018 | 0.961 |
| rs9616906 | A | G | 0.452 | 0.015 | 0.003 | 0.000 |  | -0.003 | 0.016 | 0.848 |
| rs9739070 | A | G | 0.230 | 0.024 | 0.003 | 0.000 |  | -0.021 | 0.019 | 0.284 |
| rs9792504 | A | G | 0.694 | -0.018 | 0.003 | 0.000 |  | -0.009 | 0.017 | 0.580 |
| SNP | EA | Other | EAF | Associations with Education | | |  | Associations with Hemorrhagic stroke | | |
|  |  | allele |  | Beta | se | P value |  | Beta | se | P value |
| rs10006235 | T | C | 0.287 | -0.015 | 0.003 | 0.000 |  | 0.000 | 0.000 | 0.840 |
| rs1008078 | T | C | 0.373 | -0.016 | 0.003 | 0.000 |  | 0.000 | 0.000 | 0.560 |
| rs1035578 | A | G | 0.569 | -0.013 | 0.002 | 0.000 |  | 0.000 | 0.000 | 0.150 |
| rs10483349 | A | G | 0.830 | -0.019 | 0.003 | 0.000 |  | 0.000 | 0.000 | 0.810 |
| rs10772644 | C | G | 0.871 | 0.021 | 0.004 | 0.000 |  | 0.000 | 0.000 | 0.660 |
| rs10831912 | T | C | 0.403 | -0.015 | 0.003 | 0.000 |  | 0.000 | 0.000 | 0.920 |
| rs1106761 | A | G | 0.360 | -0.017 | 0.003 | 0.000 |  | 0.000 | 0.000 | 0.330 |
| rs11130222 | A | T | 0.577 | 0.026 | 0.003 | 0.000 |  | 0.000 | 0.000 | 0.280 |
| rs111321694 | T | C | 0.183 | -0.018 | 0.003 | 0.000 |  | 0.000 | 0.000 | 0.028 |
| rs11191193 | A | G | 0.651 | 0.019 | 0.003 | 0.000 |  | 0.000 | 0.000 | 0.400 |
| rs11222416 | T | C | 0.416 | -0.015 | 0.003 | 0.000 |  | 0.000 | 0.000 | 0.890 |
| rs11588857 | A | G | 0.209 | 0.022 | 0.003 | 0.000 |  | 0.000 | 0.000 | 0.050 |
| rs11687170 | T | C | 0.828 | 0.024 | 0.004 | 0.000 |  | 0.000 | 0.000 | 0.820 |
| rs11726992 | T | C | 0.646 | 0.014 | 0.003 | 0.000 |  | 0.000 | 0.000 | 0.660 |
| rs12410444 | A | G | 0.718 | -0.018 | 0.003 | 0.000 |  | 0.000 | 0.000 | 0.420 |
| rs12514965 | T | C | 0.739 | 0.018 | 0.003 | 0.000 |  | 0.000 | 0.000 | 0.030 |
| rs12534506 | A | T | 0.453 | -0.015 | 0.003 | 0.000 |  | 0.000 | 0.000 | 0.810 |
| rs12761761 | T | C | 0.207 | 0.017 | 0.003 | 0.000 |  | 0.000 | 0.000 | 0.190 |
| rs12900061 | A | G | 0.162 | 0.021 | 0.003 | 0.000 |  | 0.000 | 0.000 | 0.480 |
| rs12962421 | A | G | 0.537 | -0.014 | 0.002 | 0.000 |  | 0.000 | 0.000 | 0.770 |
| rs12969294 | A | G | 0.379 | -0.018 | 0.003 | 0.000 |  | 0.000 | 0.000 | 0.390 |
| rs12987662 | A | C | 0.379 | 0.022 | 0.003 | 0.000 |  | 0.000 | 0.000 | 0.460 |
| rs13010288 | T | G | 0.112 | 0.020 | 0.004 | 0.000 |  | 0.000 | 0.000 | 0.940 |
| rs13421974 | T | C | 0.522 | 0.014 | 0.002 | 0.000 |  | 0.000 | 0.000 | 0.570 |
| rs1378214 | T | C | 0.383 | -0.016 | 0.003 | 0.000 |  | 0.000 | 0.000 | 0.450 |
| rs1382358 | T | C | 0.907 | 0.021 | 0.004 | 0.000 |  | 0.000 | 0.000 | 0.860 |
| rs1396967 | T | C | 0.606 | -0.015 | 0.003 | 0.000 |  | 0.000 | 0.000 | 0.095 |
| rs141979783 | T | C | 0.052 | 0.037 | 0.006 | 0.000 |  | 0.000 | 0.000 | 0.650 |
| rs1424580 | T | C | 0.800 | 0.018 | 0.003 | 0.000 |  | 0.000 | 0.000 | 0.030 |
| rs152590 | C | G | 0.343 | 0.014 | 0.003 | 0.000 |  | 0.000 | 0.000 | 0.400 |
| rs16845580 | T | C | 0.631 | 0.016 | 0.003 | 0.000 |  | 0.000 | 0.000 | 0.140 |
| rs17425572 | A | G | 0.440 | 0.014 | 0.002 | 0.000 |  | 0.000 | 0.000 | 0.140 |
| rs17824247 | T | C | 0.580 | -0.018 | 0.003 | 0.000 |  | 0.000 | 0.000 | 0.300 |
| rs2456973 | A | C | 0.679 | -0.018 | 0.003 | 0.000 |  | 0.000 | 0.000 | 0.100 |
| rs28420834 | A | G | 0.429 | -0.015 | 0.003 | 0.000 |  | 0.000 | 0.000 | 0.170 |
| rs28792186 | T | C | 0.605 | -0.025 | 0.003 | 0.000 |  | 0.000 | 0.000 | 0.690 |
| rs3095075 | A | G | 0.550 | -0.014 | 0.002 | 0.000 |  | 0.000 | 0.000 | 0.730 |
| rs320700 | A | G | 0.634 | 0.016 | 0.003 | 0.000 |  | 0.000 | 0.000 | 0.920 |
| rs34305371 | A | G | 0.088 | 0.036 | 0.004 | 0.000 |  | 0.000 | 0.000 | 0.840 |
| rs34344888 | A | G | 0.397 | -0.016 | 0.003 | 0.000 |  | 0.000 | 0.000 | 0.150 |
| rs35771425 | T | C | 0.795 | 0.019 | 0.003 | 0.000 |  | 0.000 | 0.000 | 0.190 |
| rs4240470 | C | G | 0.707 | 0.016 | 0.003 | 0.000 |  | 0.000 | 0.000 | 0.680 |
| rs4244613 | A | G | 0.416 | -0.014 | 0.003 | 0.000 |  | 0.000 | 0.000 | 0.170 |
| rs4468571 | A | G | 0.577 | -0.014 | 0.003 | 0.000 |  | 0.000 | 0.000 | 0.940 |
| rs4478846 | T | C | 0.853 | 0.018 | 0.003 | 0.000 |  | 0.000 | 0.000 | 0.970 |
| rs4493682 | C | G | 0.203 | 0.019 | 0.003 | 0.000 |  | 0.000 | 0.000 | 0.082 |
| rs4800490 | A | C | 0.543 | -0.015 | 0.002 | 0.000 |  | 0.000 | 0.000 | 0.630 |
| rs4863692 | T | G | 0.334 | 0.018 | 0.003 | 0.000 |  | 0.000 | 0.000 | 0.950 |
| rs4974424 | A | G | 0.827 | -0.019 | 0.003 | 0.000 |  | 0.000 | 0.000 | 0.980 |
| rs523934 | A | G | 0.416 | 0.015 | 0.003 | 0.000 |  | 0.000 | 0.000 | 0.160 |
| rs538628 | C | G | 0.244 | -0.018 | 0.003 | 0.000 |  | 0.000 | 0.000 | 0.250 |
| rs58694847 | C | G | 0.310 | -0.018 | 0.003 | 0.000 |  | 0.000 | 0.000 | 0.730 |
| rs61160187 | A | G | 0.619 | -0.018 | 0.003 | 0.000 |  | 0.000 | 0.000 | 0.400 |
| rs62100767 | A | G | 0.601 | 0.014 | 0.003 | 0.000 |  | 0.000 | 0.000 | 0.350 |
| rs62263923 | A | G | 0.644 | -0.016 | 0.003 | 0.000 |  | 0.000 | 0.000 | 0.310 |
| rs6839705 | A | C | 0.360 | 0.017 | 0.003 | 0.000 |  | 0.000 | 0.000 | 0.770 |
| rs6882046 | A | G | 0.687 | -0.021 | 0.003 | 0.000 |  | 0.000 | 0.000 | 0.120 |
| rs7029201 | A | G | 0.424 | 0.025 | 0.003 | 0.000 |  | 0.000 | 0.000 | 0.360 |
| rs7033137 | C | G | 0.774 | 0.016 | 0.003 | 0.000 |  | 0.000 | 0.000 | 0.780 |
| rs7146434 | A | G | 0.565 | -0.014 | 0.002 | 0.000 |  | 0.000 | 0.000 | 0.860 |
| rs71537331 | T | C | 0.371 | -0.017 | 0.003 | 0.000 |  | 0.000 | 0.000 | 0.046 |
| rs7599488 | T | C | 0.425 | -0.017 | 0.002 | 0.000 |  | 0.000 | 0.000 | 0.160 |
| rs766406 | T | G | 0.619 | 0.014 | 0.003 | 0.000 |  | 0.000 | 0.000 | 0.450 |
| rs7757476 | A | G | 0.231 | 0.020 | 0.003 | 0.000 |  | 0.000 | 0.000 | 0.480 |
| rs7948975 | T | C | 0.659 | 0.014 | 0.003 | 0.000 |  | 0.000 | 0.000 | 0.680 |
| rs7964899 | A | G | 0.457 | 0.017 | 0.002 | 0.000 |  | 0.000 | 0.000 | 0.420 |
| rs8049439 | T | C | 0.655 | 0.015 | 0.003 | 0.000 |  | 0.000 | 0.000 | 0.340 |
| rs9527702 | A | G | 0.763 | 0.023 | 0.003 | 0.000 |  | 0.000 | 0.000 | 0.370 |
| rs9556958 | T | C | 0.502 | -0.015 | 0.002 | 0.000 |  | 0.000 | 0.000 | 0.570 |
| rs9616906 | A | G | 0.452 | 0.015 | 0.003 | 0.000 |  | 0.000 | 0.000 | 0.690 |
| rs9739070 | A | G | 0.230 | 0.024 | 0.003 | 0.000 |  | 0.000 | 0.000 | 0.740 |
| rs9792504 | A | G | 0.694 | -0.018 | 0.003 | 0.000 |  | 0.000 | 0.000 | 0.410 |

EA, Effect allele; EAF, Effect allele frequency; se, Standard error; SNP, Single-nucleotide polymorphism.
