# Supplementary material for: Education and stroke: evidence from epidemiology and Mendelian randomization study
Source: Sci Rep. 2020 Dec 3;10:21208. doi: 10.1038/s41598-020-78248-8 (PMC7713498; doi:10.1038/s41598-020-78248-8)
Supplement: Supplementary file 4 — Supplementary Table 2. [file 41598_2020_78248_MOESM4_ESM.docx]

Table Supplement 2 Details of studies and datasets used for analyses

| Exposure/Outcomes | Participants | Sample size | ID/Pubmed ID | First author | Consortium | Year | Units |
| --- | --- | --- | --- | --- | --- | --- | --- |
| Education | European, Males and Females | 293723 | 27225129 | Okbay | SSGAC | 2016 | SD (3.6 years) |
| Stroke | European, Males and Females | 463010 | UKB-b:6813 | Ben Elsworth | MRC-IEU | 2018 | NA |
| Ischemic stroke | Mixed, Males and Females | 29633 | 26935894 | Malik | ISGC | 2016 | NA |
| Hemorrhagic stroke | Mixed, Males and Females | 463010 | UKB-b:4538 | Ben Elsworth | MRC-IEU | 2018 | NA |

NA, not available.
